# Level‐specific reliability coefficients from the perspective of latent state‐trait theory

**DOI:** 10.1111/bmsp.70027

**Published:** 2025-12-27

**Authors:** Lennart Nacke, Axel Mayer

**Affiliations:** ^1^ Department of Psychological Methods and Evaluation Faculty of Psychology and Sport Science, Bielefeld University Bielefeld Germany

**Keywords:** intensive longitudinal data, latent state‐trait theory, multilevel latent state‐trait models, narcissism, reliability

## Abstract

The growing popularity of the ecological momentary assessment method in psychological research requires adequate statistical models for intensive longitudinal data (ILD), with multilevel latent state‐trait (ML‐LST) models based on the latent state‐trait theory revised (LST‐R theory) as one possible alternative. Besides the traditional LST‐R coefficients reliability, consistency and occasion‐specificity, ML‐LST models are also suitable for estimating reliability at Level 1 (“within‐subject reliability”) and Level 2 (“between‐subject reliability”). However, these level‐specific coefficients have not yet been defined in LST‐R theory and, therefore, their interpretation has been unclear from the perspective of LST‐R theory. In the current study, we discuss the interpretation and identification of these coefficients based on the (multilevel) versions of the Multistate‐Singletrait (MSST), the Multistate‐Indicator‐specific trait (MSIT)  and the Multistate‐Singletrait model with M‐1 correlated method factors (MSST‐M‐1). We show that, in the MSST‐M‐1 model, the between‐subject coefficient is a measure of the indicator‐unspecificity of an item (i.e. the portion of between‐level variance that a specific item shares with a common trait) or the unidimensionality of a scale. Moreover, we highlight differences between occasion‐specificity and within‐subject reliability. The performance of the ML‐MSST‐M‐1 model and the corresponding theoretical findings are illustrated using data from an experience sampling study on the within‐person fluctuations of narcissistic admiration (Heyde et al., 2023).

## 1 INTRODUCTION

In the social sciences, researchers frequently deal with the analysis of longidutinal data, often with the specific aim to disentangle within‐person processes from rather stable, trait‐like properties. This differentiation can be important when evaluating the reliability of a psychometric test or questionnaire that has been applied in a longitudinal study. For example, a researcher might not only be interested in the reliability of a measure, but rather how reliable the respective instrument estimates trait‐like or state‐specific properties. There are several statistical models that have been developed for this purpose (for an overview, see Castro‐Alvarez et al., 2026), with models based on the Latent State‐Trait Theory Revised (LST‐R theory; Steyer et al., 2015) as a well‐known alternative. In LST‐R theory, the variance of an observed variable $Y_{it}$ is decomposed into variance due to trait‐specific influences, situational factors and person‐situation interactions, as well as measurement error. This allows for the decomposition of the total reliability of a measure into two components: the consistency coefficients $Con(Y_{it})$, estimating the proportion of observed variance that is due to (largely) stable, trait‐specific influences, and the occasion‐specificity coefficient $OSpe(Y_{it})$, accounting for the share of the observed variance that is due to situational influences and person‐situation interactions. Several structural equation models have been developed based on the LST‐R theory to estimate these coefficients. Due to obstacles that are commonly associated with intensive longitudinal data (ILD; e.g. unequally spaced time points between individuals, a large number of measurement occasions; Castro‐Alvarez et al., 2022; Geiser et al., 2013), multilevel LST models (ML‐LST models) have been developed as an alternative to traditional LST models (which require a wide‐formatted data set). While these models can be used to estimate reliability, consistency, and occasion‐specificity in ESM data, they are also capable of estimating level‐specific coefficients (i.e. Level‐1 and Level‐2 reliability coefficients) that have been termed within‐ and between‐subject reliability in the literature not explicitly referring to LST‐R theory (Geldhof et al., 2014).^1^ However, these level‐specific coefficients are neither reported in ML‐LST analyses nor defined in LST‐R theory as they have been originally developed in a statistical framework based on multilevel factor analysis (ML‐CFA; Geldhof et al., 2014). Furthermore, the ML‐CFA models that are used to estimate these coefficients (e.g. Frumkin et al., 2023; Heyde et al., 2023) are not in line with LST‐R theory since they contain orthogonal method factors at level 2 that cannot be defined as conditional expectations of observed variables (e.g. Geiser & Lockhart, 2012; see also Appendix A, Figure A1), thereby obscuring the interpretation of the resulting between‐subject coefficient. In the current study, we address this issue by defining level‐specific coefficients based on an LST‐R perspective. Additionally, we introduce the ML‐LST model with M‐1 method factors (MSST‐M‐1 model), which is a multilevel version of the established LST model with M‐1 method factors (Eid & Diener, 1999; Eid, 2000). This model enables researchers to estimate within‐ and between‐subject coefficients that can be meaningfully interpreted (both item‐ and scale‐wise): the within‐subject reliability coefficient measuring the reliability of within‐person fluctuations around a person‐specific mean or trait (e.g. Brose et al., 2020; Nezlek, 2017), and the between‐subject coefficient corresponding to the indicator unspecificity of an item (e.g. Koch & Semmler‐Busch, 2018; Eid et al., 2025) or the unidimensionality (Geiser & Lockhart, 2012) of an entire scale. The next section gives an overview of the basic variables and coefficients LST‐R theory (Steyer et al., 2015) and introduces formal definitions of the within‐ and the between‐subject coefficients. Afterwards, the corresponding (ML)‐LST models and their assumptions are presented in more detail, with a particular focus on the LST‐R and level‐specific coefficients that can be estimated with each model. The presentation of each model is accompanied by an empirical example—a data set that has been obtained as part of an ESM study of the situational fluctuations of narcissistic admiration (Heyde et al., 2023)—to illustrate the meaning of the LST‐R and level‐specific coefficients.

## 2 A SHORT INTRODUCTION TO LST‐R THEORY

Based on the assumption that a single trait measurement never occurs in a situational vacuum (Steyer et al., 2015), LST‐R theory extends the assumptions of Classical Test Theory (CTT; Lord & Novick, 1968) for the analysis of longitudinal data. In CTT, an observed variable $Y_{i}$ containing quantifiable answers from a given set of persons on an item i can be decomposed into a true score variable $\tau_{i}$ and a measurement error variable $\varepsilon_{i}$:

(1) $$\begin{array}{l} Y_{i}=\tau_{i}+\varepsilon_{i} \end{array}$$

The main idea is that situational factors influencing the true score variable $\tau_{i}$ can be distinguished from trait‐like sources of individual differences using a longitudinal research design. In particular, the index t is added to the true score variable $\tau_{i}$ which is then called latent state variable $\tau_{it}$ and comprises trait‐specific influences, as well as influences due to situational influences and person‐situation interactions. The latent state variable $\tau_{it}$ can, thus, be further divided into a latent trait variable $\xi_{it}$ and a latent state residual variable $\zeta_{it}$:

(2) $$\begin{array}{l} Y_{it}=\tau_{it}+\varepsilon_{it} \end{array}$$

(3) $$\begin{array}{l} \tau_{it}=\xi_{it}+\zeta_{it} \end{array}$$

Based on this decomposition, the reliability of an observed variable $Y_{it}$ can be further compartmentalized into the consistency coefficient (the trait‐specific proportion of reliability) and the occasion‐specificity coefficient (the state‐residual‐specific proportion of reliability). As in CTT, the reliability of an observed variable $Y_{it}$ is defined as the ratio of the true score variance $Var(\tau_{it})$ to the overall variance $Var(Y_{it})$ (which itself is given by the sum of the variances of the latent trait, latent state residual and measurement error variables; $Var(\xi_{it})+Var(\zeta_{it})+Var(\varepsilon_{it})$):

(4) $$\begin{array}{l} Rel(Y_{it}):=\frac{Var(\tau_{it})}{Var(Y_{it})}=\frac{Var(\xi_{it})+Var(\zeta_{it})}{Var(\xi_{it})+Var(\zeta_{it})+Var(\varepsilon_{it})} \end{array}$$

Due to the aforementioned compartmentalization of the true score variable $\tau_{it}$, the reliability of a measure can be likewise divided into trait‐ and state‐residual‐specific components. The consistency coefficient $Con(Y_{it})$ quantifies how well (on average) the observed variable $Y_{it}$ estimates the latent trait variable $\xi_{it}$, whereas the occasion‐specificity coefficient $OSpe(Y_{it})$ gives the proportion of the variance of $Y_{it}$ that is accounted for by the latent state residual variable $\zeta_{it}$ (Steyer et al., 2015):

(5) $$\begin{array}{l} Con(Y_{it}):=\frac{Var(\xi_{it})}{Var(Y_{it})}=\frac{Var(\xi_{it})}{Var(\xi_{it})+Var(\zeta_{it})+Var(\varepsilon_{it})} \end{array}$$

(6) $$\begin{array}{l} OSpe(Y_{it}):=\frac{Var(\zeta_{it})}{Var(Y_{it})}=\frac{Var(\zeta_{it})}{Var(\xi_{it})+Var(\zeta_{it})+Var(\varepsilon_{it})} \end{array}$$

From the Equations ((4), (5), (6)), it follows that the reliability coefficient is always the sum of consistency and occasion‐specificity:

(7) $$\begin{array}{l} Rel(Y_{it}):=Con(Y_{it})+OSpe(Y_{it}) \end{array}$$

In empirical applications, estimates of these coefficients can be used to assess if a given measure actually captures the amount of trait‐ or state‐residual‐specific variance that it intends to. For example, if a questionnaire that has been designed to measure trait anxiety (in contrast to state‐anxiety; Spielberger, 1972) is repeatedly administered to the same sample, the variance of each item should be predominantly explained by trait‐specific influences, resulting in larger consistency coefficients and a smaller occasion‐specificity coefficients for these items. Conversely, a questionnaire measuring state anxiety should primarily consist of items with larger occasion‐specificity and lower consistency.

### 2.1 Level‐specific reliability coefficients in LST‐R theory

As the LST‐R approach is based on a longitudinal design, meaning that the units of analysis are hierarchically structured (i.e. measurement occasions at Level 1 are nested within persons at Level 2), reliability coefficients can, in addition to the traditional LST‐R coefficients reliability, consistency and occasion‐specificity, be defined for Level 1 (the within‐person level) and Level 2 (the between‐person level) separately. Geldhof et al. (2014) introduced an ML‐CFA‐based approach for estimating level‐specific reliability coefficients in (cross‐sectional and longitudinal) multilevel designs, but, to our knowledge, there is no literature providing formal (model‐free) definitions of these coefficients. In longitudinal multilevel designs, the $Y_{it}$ variables can be decomposed into within‐ and between‐person components, $Y_{it}=Y_{it}^{w}+Y_{it}^{b}$ (Geldhof et al., 2014; Lai, 2021), where $Y_{it}^{w}=\zeta_{it}+\varepsilon_{it}$ and $Y_{it}^{b}=E(Y_{it}|U_{t})=\xi_{it}$ (Geiser et al., 2013). The $Y_{it}^{w}$ variable, therefore, represents deviations from the latent trait variable $\xi_{it}$, $Y_{it}^{w}=Y_{it}-\xi_{it}$. The reliability coefficient at the within‐level $Rel^{w}(Y_{it})$ can then be considered as the reliability of $Y_{it}^{w}$, which is given by the ratio of the variance of the latent state residual variable $\zeta_{it}$ and the variance of the within‐level variable $Y_{it}^{w}$ (Geldhof et al., 2014; Lai, 2021):

(8) $$\begin{array}{l} Rel^{w}(Y_{it}):=\frac{Var(\zeta_{it})}{Var(Y_{it}^{w})}=\frac{Var(\zeta_{it})}{Var(\zeta_{it})+Var(\varepsilon_{it})} \end{array}$$

Note that the $Rel^{w}(Y_{it})$ coefficient differs from the occasion‐specificity coefficient, which additionally considers the variance of the latent trait variable $Var(\xi_{it})$ in its denominator (see Equation 6), thereby quantifying the proportion of the total variance of an observed variable $Y_{it}$ that is determined by the latent state residual variable $\zeta_{it}$ (Steyer et al., 2015). The within‐subject‐reliability coefficient as defined as in Equation (8), on the other hand, gives the proportion of the within‐level variance that is determined by latent state‐residual variable $\zeta_{it}$. In terms of LST‐R theory, it can be understood as an index that quantifies how reliably within‐person fluctations are measured by the latent state residual variable $\zeta_{it}$ (see also Hachenberger et al., 2025). This differentiation between specificity and within‐subject reliability is conceptually important when comparing specificity and within‐subject reliability coefficients differing considerably in magnitude. For example, consider a highly reliable psychological measure like a comprehensive intelligence test battery that is repeatedly administered to a large number of people. When calculating reliability, consistency, and occasion‐specificity for a single measurement occasion at the scale level, the majority of the overall variance of the total scale is most likely determined by influences due to the latent trait variable. In turn, contributions accounted for by the latent state residual variable, as well as the measurement error variable, will probably be small, resulting in high consistency and low occasion‐specificity. In this case, however, estimates of within‐subject reliability can still be large. As the within‐subject reliability coefficient is defined as the ratio of $Var(\zeta_{it})$ to the within‐level variance $Var(\zeta_{it})+Var(\varepsilon_{it})$ (see Equation 8), smaller values of $Var(\varepsilon_{it})$ result in higher estimates for the $Rel^{w}$ coefficient even if $Var(\zeta_{it})$ is comparatively low. Estimates of within‐subject reliability can, therefore, be quite substantial even in the presence of low occasion‐specificity (as we will also demonstrate in our empirical example).

Conversely, the between‐subject coefficient can be conceptualized as the ratio of the “reliable” between‐person variance to the overall between‐level variance (Geldhof et al., 2014). In terms of LST‐R theory, it can be understood as a measure of the indicator unspecificity of an item, $IUSpe(Y_{it})$; that is, the portion of between‐level variance that this item shares with a common latent trait variable. The indicator‐unspecificity coefficient can be obtained by dividing the variance of the latent trait variable $\xi_{it}$ by the total variance at the between‐subject level, including measurement error. However, since the latent trait variables $\xi_{it}$ are the only between‐level variables in LST‐R theory (as they are assumed to be free of any measurement error; Steyer et al., 2015), there are no between‐subject error variables, implying that the indicator‐unspecificity is always 1:

(9) $$\begin{array}{l} IUSpe(Y_{it}):=\frac{Var(Y_{it}^{b})}{Var(Y_{it}^{b})}=\frac{Var(\xi_{it})}{Var(\xi_{it})}=1 \end{array}$$

Conversely, substracting this coefficient from 1 yields the indicator‐specificity coefficient (e.g. Koch & Semmler‐Busch, 2018; Eid et al., 2025), which is equivalent to between‐level variance that a specific indicator i does not share with the common trait factor:

(10) $$\begin{array}{l} ISpe(Y_{it}):=1-IUSpe(Y_{it})=0 \end{array}$$

From an LST‐R perspective, both coefficients are, thus, not informative as their values are always the same and provide no additional information that could be useful in empirical applications. However, since ML‐CFA models allow for the inclusion of error variables at the between‐level, estimates for model‐based between‐subject reliability coefficients as defined by Geldhof et al. (2014) can nonetheless assume values varying between 0 and 1 (which are typically very high at >.9 when obtained in empirical studies using ILD; e.g., Frumkin et al., 2023; Heyde et al., 2023; Hachenberger et al., 2025). We will refer back to this point later when introducing the ML‐MSST‐M‐1 model, which is an LST‐R model that allows for the estimation of indicator‐(un)specificity coefficients^2^ that can be meaningfully interpreted.

## 3 (MULTILEVEL) LST MODELS AND THEIR APPLICATION TO REAL DATA

The aforementioned definitions imply item‐ and occasion‐specific latent state, trait and state residual variables. However, when analysing a real data set, means, (co)variances and factor loadings cannot be estimated without making equivalence assumptions about each latent variable (for LST models in an experience sampling context, see, for example, Norget et al., 2025). Different types of equivalence assumptions can be made leading to various LST models that have been developed to estimate the parameters of interest of the respective LST‐R variables or the LST‐R coefficients. In the following sections, we introduce three (ML‐)LST models and illustrate the meaning of the LST‐R coefficients based on a real ESM data set. In particular, we analyse data from Heyde et al. (2023) who conducted an experience sampling study on the situational fluctuations of narcissistic admiration (i.e. the tendency to maintain a grandiose self‐concept by means of self‐promotion) and rivalry (i.e. maintaining a grandiose self‐concept by means of antagonistic behaviour like devaluating others; Back et al., 2013). The sample of this study consists of 114 working adults (69.20% female; mean age = 36.81 years, SD = 12.79; for more details on the sample's composition, see Heyde et al., 2023), who provided self‐reports on various personality state measures two times per day over a fortnight (excluding the Saturdays and Sundays), resulting in a total number of 20 measurement occasions. On average, participants provided answers on 11.46 occasions, implying a response rate of 57.30% and a total number of 1306 observations. Both dimensions of narcissism were assessed with a six‐item questionnaire, each consisting of adjectives assessing admiration (e.g. “Glorious”) and rivalry (e.g. “Contemptuous”). Participants were instructed to indicate how they feel “right now” about these adjectives on a Likert scale ranging from 1 (“disagree strongly”) to 7 (“agree strongly”). For more details on the distribution of the admiration and rivalry variables, see Table 1.

**TABLE 1:** Distribution of the admiration and rivalry variables in the sample that has been obtained by Heyde et al. (2023).

$Y_{i}$	Admiration	Mean	Variance	SD	Rivalry	Mean	Variance	SD
**Scale**	**ADM**	**14.217**	**46.691**	**6.833**	**RIV**	**9.405**	**21.039**	**4.586**
$Y_{1}$	Glorious	2.709	2.377	1.542	Contemptuous	1.413	.730	.854
$Y_{2}$	Envied	1.675	1.094	1.046	Irritated	2.120	1.983	1.408
$Y_{3}$	Prestigious	2.457	2.162	1.470	Gleeful	1.406	.831	.911
$Y_{4}$	Brilliant	2.445	1.785	1.336	Envious	1.542	.959	.979
$Y_{5}$	Powerful	2.808	2.346	1.531	Resentful	1.337	.680	.824
$Y_{6}$	Superior	2.120	1.793	1.339	Angry	1.584	1.259	1.122

*Note*: ADM = Narcissistic Admiration, RIV = Narcissistic Rivalry. Means, variances, and standard deviations of the sum scores of both scales are printed in bold.

We conducted our data analysis in R 4.5.2 (R Core Team, 2025) using version 0.6‐19 of the lavaan package (Rosseel, 2012) to compute all ML‐LST models (parameter estimates were obtained based on maximum likelihood estimation). Due to the poor psychometric properties of the rivalry scale, we only use the admiration (ADM) scale, which performed much better in our preliminary analysis (see Appendix B, Table B1). We then show how the preexisting ML‐LST models—the multilevel multistate‐singletrait (ML‐MSST) model and the multilevel indicator‐specific trait (ML‐MSIT) model (Castro‐Alvarez et al., 2022; Geiser et al., 2013)—can be used to assess reliability, consistency, occasion‐specificity, and the within‐subject reliability of each item and the entire scale. Then, we introduce the multistate‐singletrait model with M‐1 correlated method factors (ML‐MSST‐M‐1 model) and show that estimates of the between‐subject coefficients that have been obtained item‐ or scale‐wise can be meaningfully interpreted as the amount of indicator‐(un)specificity.

### 3.1 (Multilevel) Multistate‐Singletrait models

In the following subsections, we refer to the first‐order formulation of LST models where trait and state residuals variables directly load on the observed variables, as these models can be specified as ML‐LST models with comparatively relaxed equivalence assumptions about the latent state residuals variables $\zeta_{it}$ and the latent trait variables $\xi_{it}$. These models were introduced by Eid (1996) and discussed in more detail by Norget et al. (2025). The main advantage of first‐order LST models is that they allow for freely varying factor loadings across levels when specified as ML‐LST models (see also Castro‐Alvarez et al., 2022; Geiser et al., 2013 who both implicitly considered the first‐order formulation when discussing the application of various ML‐LST models). In contrast, the traditional second‐order LST models (Steyer et al., 2015) make equivalence assumptions about the latent state variables $\tau_{it}$ and the latent trait variables $\xi_{it}$. This results in the necessity to assume cross‐level invariance of factor loadings in ML‐LST analyses, to ensure that the resulting ML‐LST models are equivalent to traditional second‐order LST models (which, in turn, make specific equivalence assumptions about the $\tau_{it}$ and $\xi_{it}$ variables; see also Figure 18 in the supplemental text C by Steyer et al., 2015). Relaxing the cross‐level invariance assumption of ML‐LST models and, thus, dispensing with the need to rely on the second‐order formulation, can be beneficial as it may result in an improved model fit in ML‐LST analyses.

In the first‐order (ML‐)MSST model, all $Y_{it}$ are decomposed into a common latent trait variable θ, occasion‐specific latent state residual variables $\zeta_{t}$, and occasion‐ and item‐specific measurement error variables $\varepsilon_{it}$. As elaborated above, different equivalence assumptions can be made about the $\zeta_{t}$ and θ variables (see Norget et al., 2025 for more details). In the remainder, we focus on the least restrictive equivalence assumptions, meaning $\zeta_{t}$‐ and θ‐congenericity. Furthermore, conditional mean independence (implying that measurement error variables are uncorrelated; Steyer et al., 2015) is assumed. Additionally, when specified as an ML‐LST model (Castro‐Alvarez et al., 2022; Geiser et al., 2013), the assumption of time invariance of intercepts, factor loadings and latent as well as error variances is also implicitly integrated into the model (since the t index is dropped from all variables in a long‐formatted data set, see also Figure 1b) resulting in the following equation:

(11) $$\begin{array}{l} Y_{it}=\alpha_{i}+\lambda_{Ti}\theta +\lambda_{Si}\zeta_{t}+\varepsilon_{it} \end{array}$$

with $\alpha_{i}$ representing the item‐specific intercept (with $\alpha_{1}=0)$, and $\lambda_{Ti}$ and $\lambda_{Si}$ relating the latent trait variable θ and the latent state residual variable $\zeta_{t}$ to the observed variables $Y_{it}$ (with $\lambda_{T1}$ = $\lambda_{S1}$ = 1). Note that the $\zeta_{t}$ variables have a mean of 0 and are uncorrelated since they are defined as a residual variables. The same is true for the measurement error variables $\varepsilon_{it}$. Furthermore, all intercepts $\alpha_{i}$ and loadings $\lambda_{Ti}$ and $\lambda_{Si}$ are set equal across time, ensuring that the latent trait variable only captures time‐invariant influences. Besides the necessity for these constraints in ML‐LST analyses, they are also applied to avoid a potential trait change being masked by freely varying intercepts and factor loadings (for more details, see Geiser et al., 2015).^3^ The consistency coefficient of an observed variable $Y_{it}$ can then be obtained by relating the proportion of variance accounted for by the latent trait variable θ to the overall observed variance. The occasion‐specificity coefficient, in turn, is given by dividing the variance explained by the latent state residual variable $\zeta_{t}$ by the total observed variance:

(12) $$\begin{array}{l} Con{\left(Y_{it}\right)}^{(ML-)MSST}=\frac{\lambda_{Ti}^{2}Var(\theta )}{\lambda_{Ti}^{2}Var(\theta )+\lambda_{Si}^{2}Var(\zeta_{t})+Var(\varepsilon_{it})} \end{array}$$

(13) $$\begin{array}{l} OSpe{\left(Y_{it}\right)}^{(ML-)MSST}=\frac{\lambda_{Si}^{2}Var(\zeta )}{\lambda_{Ti}^{2}Var(\theta )+\lambda_{Si}^{2}Var(\zeta_{t})+Var(\varepsilon_{it})} \end{array}$$

**FIGURE 1:**
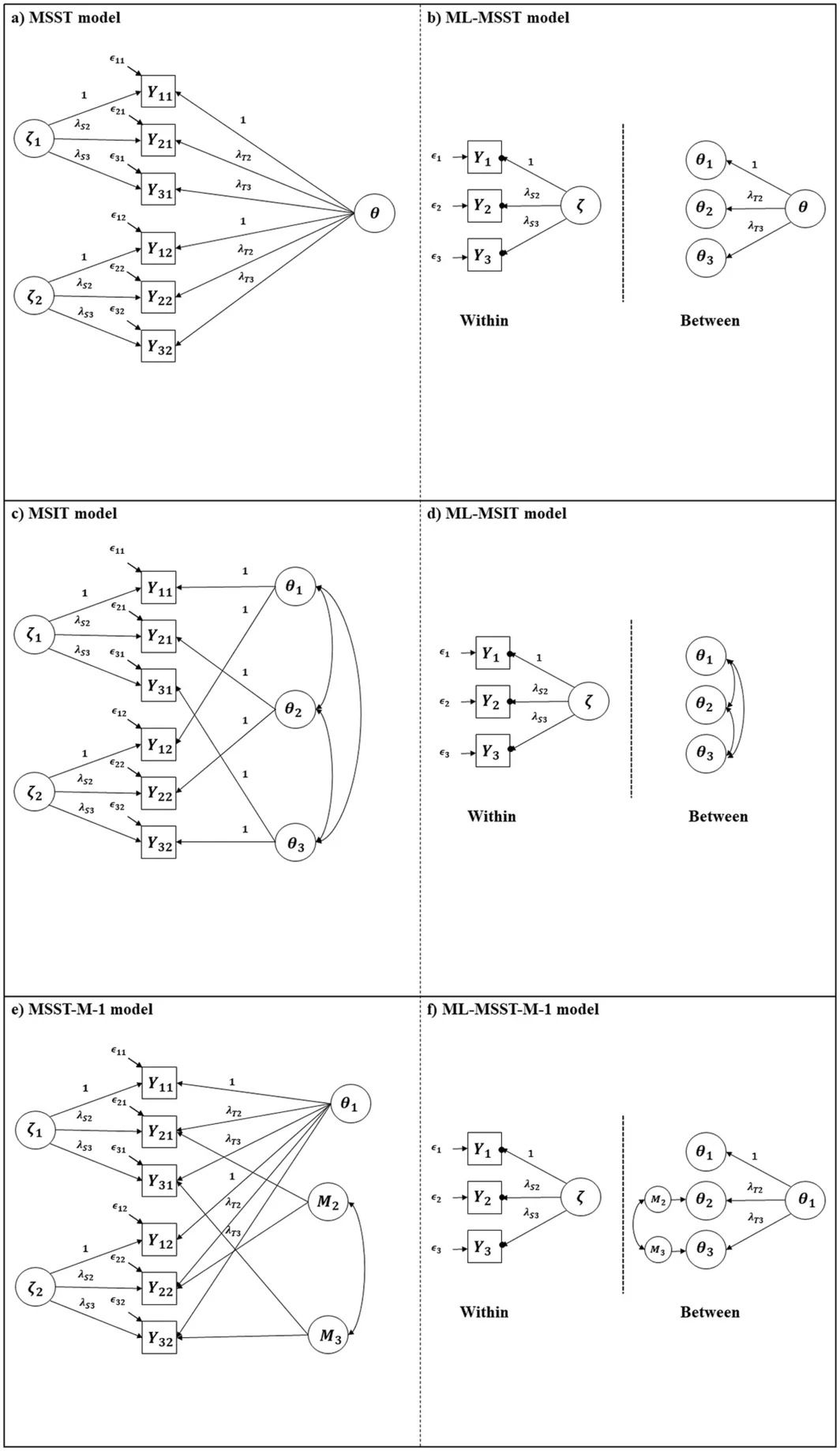
The path diagrams show each of the three (ML‐)LST models for a longitudinal design with three items that are measured at two measurement occasions. $Y_{it}$ = observed variable, θ = latent trait variable, $\zeta_{t}$ = latent state residual variable, $M_{i}$ = method factor, $\varepsilon_{it}$ = measurement error variable, $\lambda_{Ti}$= trait loading coefficient, $\lambda_{Si}$ = state residual loading coefficient, MSST = Multistate‐Singletrait, MSIT = Multistate‐Indicator‐specific trait, MSST‐M‐1 = Multistate‐Singletrait with M‐1 method factors. Loadings of the latent trait variables and latent state residual variables are invariant across time points. Invariance across time is also assumed for the intercepts (which are not shown for simplicity). The loadings of the residual variables and M‐1 method factors are fixed to 1. Variances of all latent variables and measurement error variables are also held invariant across time. The covariances between the latent variables are freely estimated.

Reliability, consistency and occasion specificity can also be obtained at the scale level (Hachenberger et al., 2025). For example, consider the (ML‐)MSST model that is described in Equation (11) applied on a repeatedly administered scale with j items (with $\lambda_{T1}=\lambda_{S1}=1$ and $\alpha_{1}=0$). For a given measurement occasion t, the sum score $S_{t}$ is decomposed as followed:

(14) $$\begin{array}{cc} S_{t} & =\overset{j}{\underset{i=1}{\sum }}Y_{it}\\ & =\overset{j}{\underset{i=1}{\sum }}\alpha_{i}+\left(\overset{j}{\underset{i=1}{\sum }}\lambda_{Ti}\right)\cdot \theta +\left(\overset{j}{\underset{i=1}{\sum }}\lambda_{Si}\right)\cdot \zeta_{t}+\overset{j}{\underset{i=1}{\sum }}\varepsilon_{it} \end{array}$$

Reliability, consistency and occasion specificity at the scale level are, thus, defined as follows:

(15) $$\begin{array}{cc} Rel{\left(S_{t}\right)}^{ML\text{‐}MSST} & =\frac{Var\left(\left(\sum_{i=1}^{j}\lambda_{Ti}\right)\cdot \theta +\left(\sum_{i=1}^{j}\lambda_{Si}\right)\cdot \zeta_{t}\right)}{Var(S_{t})}\\ & =\frac{{\left(\sum_{i=1}^{j}\lambda_{Ti}\right)}^{2}\cdot Var(\theta )+{\left(\sum_{i=1}^{j}\lambda_{Si}\right)}^{2}\cdot Var(\zeta_{t})}{Var(S_{t})} \end{array}$$

(16) $$\begin{array}{cc} Con{\left(S_{t}\right)}^{ML\text{‐}MSST} & =\frac{Var\left(\left(\sum_{i=1}^{j}\lambda_{Ti}\right)\cdot \theta \right)}{Var(S_{t})}\\ & =\frac{{\left(\sum_{i=1}^{j}\lambda_{Ti}\right)}^{2}\cdot Var(\theta )}{Var(S_{t})} \end{array}$$

(17) $$\begin{array}{cc} OSpe{\left(S_{t}\right)}^{ML\text{‐}MSST} & =\frac{Var\left(\left(\sum_{i=1}^{j}\lambda_{Si}\right)\cdot \zeta_{t}\right)}{Var(S_{t})}\\ & =\frac{{\left(\sum_{i=1}^{j}\lambda_{Si}\right)}^{2}\cdot Var(\zeta_{t})}{Var(S_{t})} \end{array}$$

Figure 1a,b shows the respective MSST model that is described in Equation (11). In Figure 1a, a classic MSST specified based on a wide‐formatted data structure is shown, whereas Figure 1b depicts a multilevel MSST (ML‐MSST) model. Note that the t index is dropped in Figure 1b since the ML‐MSST model requires long‐formatted data. As the latent state residual variables $\zeta_{t}$ reflect influences on the observed variables $Y_{it}$ due to situational circumstances and person‐situation interactions varying between measurement occasions, they are modeled at level 1, along with the measurement error variable $\varepsilon_{it}$. Conversely, the latent trait variable θ reflecting stable between‐person differences is modeled at Level 2. At the between‐level, a latent variable containing latent means of responses averaged accross all measurement occasions is computed for each item i. In the LST‐R framework, these item‐specific latent variables can be interpreted as indicator‐specific latent trait variables $\theta_{i}$ (Geiser et al., 2013). However, since the MSST model assumes a common trait variable θ completely accounting for all covariances between indicator‐specific trait variables (i.e. assuming that each item does not contain method‐specific variance across measurement occasions), the $\theta_{i}$ variables in the ML‐MSST model are modeled as deterministic functions of the common trait variable θ. As can be seen in Equation (11), an ML‐MSST model, thus, contains no additional between‐subject error variables.

This has implications for the computation of the level‐specific coefficients, which are referred to as $Rel^{w}{\left(Y_{it}\right)}^{(ML-)MSST}$, $IUSpe{\left(Y_{it}\right)}^{(ML-)MSST}$, and $ISpe{\left(Y_{it}\right)}^{(ML-)MSST}$ for the within‐subject reliability, indicator‐unspecifcity, and the indicator‐specificity coefficient, respectively. The reliability coefficient at the within level $Rel^{w}{\left(Y_{it}\right)}^{(ML-)MSST}$ can be obtained by dividing the variance accounted for by the latent state residual variable $\zeta_{t}$ by the overall within‐level variance. In contrast, the indicator‐unspecificity coefficient $IUSpe{\left(Y_{it}\right)}^{(ML-)MSST}$ is given by the ratio of the variance accounted for by the latent trait variable θ to the total between‐level variance (Geldhof et al., 2014). As the indicator‐specific trait variables at the between‐level are deterministic functions of the common latent trait variable, the indicator‐unspecificity coefficient in the ML‐MSST model $IUSpe{\left(Y_{it}\right)}^{ML-MSST}$ is always equal to 1 (see also Equation 9). Conversely, subtracting this coefficient from 1 yields the indicator‐specificity coefficient $ISpe^{ML-MSST}$, which is always 0 (see also Equation 10):

(18) $$\begin{array}{l} Rel^{w}{\left(Y_{it}\right)}^{ML-MSST}=\frac{\lambda_{Si}^{2}Var(\zeta_{t})}{\lambda_{Si}^{2}Var(\zeta_{t})+Var(\varepsilon_{i})} \end{array}$$

(19) $$\begin{array}{l} IUSpe{\left(Y_{it}\right)}^{ML-MSST}=\frac{\lambda_{Ti}^{2}Var(\theta )}{\lambda_{Ti}^{2}Var(\theta )}=1 \end{array}$$

(20) $$\begin{array}{l} ISpe{\left(Y_{it}\right)}^{ML-MSST}=1-\frac{\lambda_{Ti}^{2}Var(\theta )}{\lambda_{Ti}^{2}Var(\theta )}=0 \end{array}$$

As we have shown for the traditional LST‐R coefficients, these coefficients can also be obtained at the scale level. The corresponding formulas for $Rel^{w}(S_{t})$, $IUSpe(S_{t})$, and $ISpe(S_{t})$ can be found in Table 2, along with an overview of all other equations for the item‐ and scale‐wise LST‐R and level‐specific coefficients.

**TABLE 2:** Coefficients in (ML‐)MSST, (ML‐)MSIT and (ML‐)MSST M‐1 models.

	(ML‐)MSST	(ML‐)MSIT	(ML‐)MSST‐M‐1
*Item‐level coefficients*			
Consistency	$\frac{\lambda_{Ti}Var(\theta )}{Var(Y_{it})}$	$\frac{Var(\theta_{i})}{Var(Y_{it})}$	$\frac{\lambda_{Ti}Var(\theta_{r})+I(i\neq r)Var(M_{i})}{Var(Y_{it})}$
Occasion‐specificity	$\frac{\lambda_{Si}Var(\zeta_{t})}{Var(Y_{it})}$	$\frac{\lambda_{Si}Var(\zeta_{t})}{Var(Y_{it})}$	$\frac{\lambda_{Si}Var(\zeta_{t})}{Var(Y_{it})}$
Reliability	$Con{\left(Y_{it}\right)}^{ML-MSST}+Spe{\left(Y_{it}\right)}^{ML-MSST}$	$Con{\left(Y_{it}\right)}^{ML-MSIT}+Spe{\left(Y_{it}\right)}^{ML-MSIT}$	$Con{\left(Y_{it}\right)}^{ML-MSST-M-1}+Spe{\left(Y_{it}\right)}^{ML-MSST-M-1}$
Within‐subject reliability	$\frac{\lambda_{Si}Var(\zeta_{t})}{\lambda_{Si}Var(\zeta_{t})+Var(\varepsilon_{it})}$	$\frac{\lambda_{Si}Var(\zeta_{t})}{\lambda_{Si}Var(\zeta_{t})+Var(\varepsilon_{it})}$	$\frac{\lambda_{Si}Var(\zeta_{t})}{\lambda_{Si}Var(\zeta_{t})+Var(\varepsilon_{it})}$
Indicator‐unspecificity	$\frac{\lambda_{Ti}^{2}Var(\theta )}{\lambda_{i}^{2}Var(\theta )}=1$	$\frac{Var(\theta_{i})}{Var(\theta_{i})}=1$	$\frac{\lambda_{Ti}Var(\theta_{r})}{\lambda_{Ti}Var(\theta_{r})+I(i\neq r)Var(M_{i})}$
Indicator‐specificity	$1-\frac{\lambda_{Ti}^{2}Var(\theta )}{\lambda_{i}^{2}Var(\theta )}=0$	$1-\frac{Var(\theta_{i})}{Var(\theta_{i})}=0$	$\frac{\lambda_{Ti}(I(i\neq r)Var(M_{i})}{\lambda_{Ti}Var(\theta_{r})+I(i\neq r)Var(M_{i})}$
*Scale‐level coefficients*			
Consistency	$\frac{{\left(1+\lambda_{T2}+\lambda_{T3}\right)}^{2}Var(\theta )}{Var(S_{t})}$	$\frac{3Var(\theta_{i})+2(Cov(\theta_{1},\theta_{2})+Cov(\theta_{2},\theta_{3})+Cov(\theta_{1},\theta_{3}))}{Var(S_{t})}$	$\frac{{\left(1+\lambda_{T2}+\lambda_{T3}\right)}^{2}Var(\theta_{r})+Var(M_{2})+Var(M_{3})+2(Cov(M_{2},M_{3}))}{Var(S_{t})}$
Occasion‐specificity	$\frac{{\left(1+\lambda_{S2}+\lambda_{S3}\right)}^{2}Var(\zeta_{t})}{Var(S_{t})}$	$\frac{{\left(1+\lambda_{S2}+\lambda_{S3}\right)}^{2}Var(\zeta_{t})}{Var(S_{t})}$	$\frac{{\left(1+\lambda_{S2}+\lambda_{S3}\right)}^{2}Var(\zeta_{t})}{Var(S_{t})}$
Reliability	$Con{\left(S_{t}\right)}^{(ML-)MSST}+Spe{\left(S_{t}\right)}^{(ML-)MSST}$	$Con{\left(S_{t}\right)}^{(ML-)MSIT}+Spe{\left(S_{t}\right)}^{(ML-)MSIT}$	$Con{\left(S_{t}\right)}^{(ML-)MSST-M-1}+Spe{\left(S_{t}\right)}^{(ML-)MSST-M-1}$
Within‐subject reliability	$\frac{{\left(1+\lambda_{S2}+\lambda_{S3}\right)}^{2}Var(\zeta_{t})}{{\left(1+\lambda_{S2}+\lambda_{S3}\right)}^{2}Var(\zeta_{t})+Var(\varepsilon_{1})+Var(\varepsilon_{2})+Var(\varepsilon_{3})}$	$\frac{{\left(1+\lambda_{S2}+\lambda_{S3}\right)}^{2}Var(\zeta_{t})}{{\left(1+\lambda_{S2}+\lambda_{S3}\right)}^{2}Var(\zeta_{t})+Var(\varepsilon_{1})+Var(\varepsilon_{2})+Var(\varepsilon_{3})}$	$\frac{{\left(1+\lambda_{S2}+\lambda_{S3}\right)}^{2}Var(\zeta_{t})}{{\left(1+\lambda_{S2}+\lambda_{S3}\right)}^{2}Var(\zeta_{t})+Var(\varepsilon_{1})+Var(\varepsilon_{2})+Var(\varepsilon_{3})}$
Unidimensionality	$\frac{{\left(1+\lambda_{T2}+\lambda_{T3}\right)}^{2}Var(\theta )}{{\left(1+\lambda_{T2}+\lambda_{T3}\right)}^{2}Var(\theta )}=1$	$\frac{3Var(\theta_{i})+2(Cov(\theta_{1},\theta_{2})+Cov(\theta_{1},\theta_{3})+Cov(\theta_{2},\theta_{3}))}{3Var(\theta_{i})+2(Cov(\theta_{1},\theta_{2})+Cov(\theta_{1},\theta_{3})+Cov(\theta_{2},\theta_{3}))}=1$	$\frac{{\left(1+\lambda_{T2}+\lambda_{T3}\right)}^{2}Var(\theta_{r})}{{\left(1+\lambda_{T2}+\lambda_{T3}\right)}^{2}Var(\theta_{r})+Var(M_{2})+Var(M_{3})+2(Cov(M_{2},M_{3}))}$

*Note*: (ML)‐MSST = (Multilevel) Multistate‐Singletrait, (ML‐)MSIT = (Multilevel) Multistate‐Indicator‐specific‐trait, (ML‐)MSST‐M‐1 = (Multilevel) reference indicator specification. Equations show model‐specific forms of consistency, specificity, and reliability, as well as within‐subject reliability and indicator‐specificity at both the item‐ and the scale‐level. Scale‐level formulas are based on three items, but the number of items could be extended ad libitum. I(i≠r) is an indicator variable with I(i≠r)=1 if i≠r and I(i≠r)=0 if i=r.

In empirical applications, the assumption of a single common trait variable θ is usually not tenable since indicators typically share idiosyncratic variance over time or method effects (i.e. persons usually provide more similar answers to the same item over time; Geiser & Lockhart, 2012). This was also strikingly confirmed in our analysis, revealing a very poor model fit of the ML‐MSST model when applied to the ADM scale ($\chi^{2}$(24) = 2326.310, RMSEA = .246, CFI = .000, AIC = 20,381.394, BIC = 20,505.587).^4^ All estimates of the LST‐R and level‐specific coefficients can be found in Table 3. However, they cannot be meaningfully interpreted due to the poor performance of the ML‐MSST model. To overcome the obstacle of too restrictive model‐implied covariance matrices that are typically associated with empirical applications of (ML‐)MSST models, method effects capturing across‐time variance unique to each item can be considered, with the implementation of indicator‐specific trait variables as a convenient and frequently applied alternative (Eid, 1996).

### 3.2 LST models with indicator‐specific trait variables

In (ML‐)MSIT models (Eid, 1996; Geiser et al., 2013), a latent trait variable $\theta_{i}$ is modeled for each indicator i separately. Again, to specify the multilevel version of this model, different equivalence and invariance assumptions have to be made. In the following einsertquation, we specify an (ML‐)MSIT model assuming $\zeta_{t}$‐congenericity, conditional mean independence, and scale invariance over time for $\zeta_{t}$, as well as for $\theta_{i}$, resulting in the following decomposition of the observed variables $Y_{it}$:

(21) $$\begin{array}{l} Y_{it}=\alpha_{i}+\lambda_{Ti}\theta_{i}+\lambda_{Si}\zeta_{t}+\varepsilon_{it} \end{array}$$

Note that the assumption of scale invariance over time implies $\lambda_{Ti}=1$ (see Figure 1c,d). Each indicator‐specific trait variable acts as *trait‐method‐unit* (Campbell & Fiske, 1959), capturing both trait‐specific influences and influences unique to a single item over time. By modeling covariances between these indicator‐specific trait variables, the amount of indicator (un)specificity of different items can be obtained by looking at the respective correlation coefficients. For example, if the correlation between two indicator‐specific trait variables is nearly 1, the variance that two items share over time largely overlaps, indicating low indicator specificity. On the other hand, a correlation that is considerably lower than 1 hints at substantial indicator‐specific variance across time, meaning that an MSST model including a single trait variable θ likely does not fit the data well. The indicator‐specific trait approach has several advantages. For example, MSIT models usually perform equally well or even better when compared to other possibilities for capturing method effects in LST and related models (i.e. models with M uncorrelated method factors; Steyer et al., 1992, or models with indicator‐specific residuals to covary over time; Steyer & Schmitt, 1990). Furthermore, each indicator‐specific trait variable is clearly defined within LST‐R theory as a conditional expectation of an observed variable (Eid, 1996; Geiser & Lockhart, 2012).

The computation of the consistency coefficient is slightly different in the (ML‐)MSIT model, relating the variance accounted for by a given indicator‐specific trait variable $\theta_{i}$ to the overall observed variance. The same is true for the indicator‐unspecificity and indicator‐specificity coefficients, which are computed for each indicator‐specific trait variable separately (but are always 1 and 0, respectively, as in the MSST model):

(22) $$\begin{array}{l} Con{\left(Y_{it}\right)}^{(ML-)MSIT}=\frac{Var(\theta_{i})}{Var(\theta_{i})+\lambda_{Si}^{2}Var(\zeta_{t})+Var(\varepsilon_{it})} \end{array}$$

(23) $$\begin{array}{l} IUSpe{\left(Y_{it}\right)}^{(ML-)MSIT}=\frac{Var(\theta_{i})}{Var(\theta_{i})}=1 \end{array}$$

(24) $$\begin{array}{l} ISpe{\left(Y_{it}\right)}^{(ML-)MSIT}=1-\frac{Var(\theta_{i})}{Var(\theta_{i})}=0 \end{array}$$

The formulas for all other LST‐R and level‐specific coefficients that can be obtained using the (ML‐)MSIT model are again summarized in Table 2. The traditional, wide‐formatted MSIT model assuming strong invariance across measurement occasions is shown in Figure 1c, while the corresponding ML‐MSIT model is shown in Figure 1d (both models are referred to in Equation 21). Note that the between‐level model specification of the ML‐MSIT model is rather simple, as the covariances between the item‐specific latent variables are freely estimated, meaning that additional restrictive assumptions about the model‐implied covariance matrix at the between‐level (e.g. a θ‐congeneric measurement model) are not needed.

In our empirical application, the ML‐MSIT model fared notably better than the ML‐MSST model ($\chi^{2}$(9) = 34.285, RMSEA = .046, CFI = .986, AIC = 18,152.137, BIC = 18,353.951). However, item‐wise estimates for reliability (ranging from .712 to .860), consistency (.633 to .741) and specificity (.038 to .118) were comparable. The same was true for within‐subject reliability estimates (ranging from .116 to .481 for item‐wise estimates and .730 for the entire scale). Again, all indicator unspecificity and specificity estimates were 1 and 0, respectively (as they are by definition). At the scale‐level, reliability (.949), consistency (.811) and specificity estimates (.138) were also comparable. All point estimates for the LST‐R and level‐specific coefficients are also reported in Table 2.

### 3.3 (Multilevel) Multistate‐Singletrait models with M‐1 correlated method factors

Similar to the (ML‐)MSST model, the Multistate‐Singletrait model with M‐1 correlated method factors (Eid, 2000; Eid & Diener, 1999) assumes a latent trait factor $\theta_{r}$ with loadings on each item. To account for potential method effects, the model includes M‐1 correlated method factors (with M being the total number of items or methods) that are allowed to covary, meaning that, for M items, M‐1 method factors are included. The item without an additional method factor is considered as a reference or “gold standard” item. As a consequence, the latent trait factor $\theta_{r}$ comprises trait‐specific influences of all items (without the idiosyncratic, method‐specific contributions of the M‐1 items), as well as method‐specific variance of the reference item. This is the reason why the latent trait factor $\theta_{r}$ is usually referred to as reference trait factor (Geiser & Lockhart, 2012). The model equation of the (ML‐)MSST M‐1 model (see also Figure 1e,f) is the following:

(25) $$\begin{array}{l} Y_{it}=\alpha_{it}+\lambda_{Ti}\theta_{r}+I(i\neq r)M_{i}+\lambda_{Si}\zeta_{t}+\varepsilon_{it} \end{array}$$

where $\alpha_{i}$ is the intercept (with $\alpha_{1}=0$), $\theta_{r}$ pertains to the trait reference factor, $\lambda_{Ti}$ and $\lambda_{Si}$ are the loading coefficients relating the trait reference factor $\theta_{r}$ and the latent state residual variable $\zeta_{t}$ to the observed variables $Y_{it}$ (with $\lambda_{T1}=\lambda_{S1}$ = 1), $M_{i}$ is a method factor pertaining to the respective non‐reference item i (whereby all loading coefficients of the non‐reference items are fixed to 1),^5^ and I(i≠r) is an indicator variable with I(i≠r)=1 if i≠r and I(i≠r)=0 if i=r. The (ML‐)MSST‐M‐1 model in Equation (25) is a reformulation of the (ML)‐MSIT model, meaning that it provides the same model fit values when applied to the same data set. As a consequence, each variable of this model can be defined as a conditional expectation of observed or other latent variables (see Geiser & Lockhart, 2012, Appendix A for additional information), meaning that this model is consistent with LST‐R theory. From our point of view, the main advantage of the ML‐MSST‐M‐1 model when compared to the ML‐MSST and ML‐MSIT models is that it provides interpretable estimates for the within‐ and between‐level coefficients—while the within‐subject reliability estimates are always the same as in the ML‐MSIT model (as they are reformulations from each other imposing the same model‐implied covariance structure at the within‐level), each model may produce different estimates at the between‐level. As the ML‐MSST‐M‐1 model allows for the inclusion of M‐1 between subject error variables at the between‐level (one indicator‐specific residual variable for each non‐reference item), the indicator‐(un)specificity estimates of the non‐reference items may be between 0 and 1 as they quantify the proportion of the variance that is (not) unique to each non‐reference item. For the entire scale, the between‐subject coefficient quantifies the amount of the between‐level variance that is accounted for by the reference trait variable $\theta_{r}$. In this case, the between‐subject coefficient can be interpreted as the amount of unidimensionality of the entire scale (with larger values implying higher unidimensionality). From our point of view, it is valuable to consider the ML‐MSST‐M‐1 model when estimating the between‐subject coefficient of the (non‐reference) items or the entire scale as other ML‐CFA‐based approaches (Geldhof et al., 2014; Lai, 2021) rely on models with orthogonal method (OM) factors at the between level. For example, Geldhof et al. (2014) proposed a method for estimating various within‐ and between‐subject coefficients based on an ML‐CFA model with uncorrelated between‐subject error variables, which can be interpreted as a multilevel version of an LST model with OM factors (Geiser & Lockhart, 2012; Steyer et al., 1992). The OM approach, however, has been discarded by Steyer and colleagues as the orthogonal method factors cannot be meaningfully defined as conditional expectations of observed variables or other latent variables (Eid, 1996; Geiser & Lockhart, 2012). Therefore, applying the ML‐MSST‐M‐1 model may be beneficial to researchers since this model is capable of providing estimates for reliability, consistency, occasion specificity, as well as within‐ and between‐subject coefficients that can be meaningfully interpreted.

As the ML‐MSST‐M‐1 model is a reformulation of the ML‐MSIT model, the performance of the ML‐MSST‐M‐1 LST model was identical to that of the ML‐MSIT model ($\chi^{2}$(9) = 34.285, RMSEA = .046, CFI = .986, AIC = 18,152.137, BIC = 18,353.951). The same was true for all item‐wise and scale‐wise obtained coefficients, except for the indicator (un)specificity and unidimensionality estimates as they are differently computed using this model when compared to the ML‐MSST and ML‐MSIT models. For the non‐reference items 3, 4 and 5, indicator‐specificity estimates were rather low with values ranging from .221 to .443 (see Table 3 for the specific point estimates). Item 2, however, showed a remarkably high indicator‐specificity at .696. For the entire scale, the unidimensionality was rather high at .844. For an overview of the results from the ML‐MSST‐M‐1 analysis, see again Table 3.

**TABLE 3:** Parameter estimates and fit statistics for the ML‐MSST, ML‐MSIT, and ML‐MSST M‐1 models.

ML‐MSST	Rel	Con	OSpe	$Rel^{w}$	IUSpe	ISpe	Model fit								
**Scale**	**.913**	**.804**	**.104**	**.556**	**1.0**	**.0**	$\chi^{2}$	2293.542							
$Y_{1}$	.610	.512	.098	.201	1.0	.0	p	<.001							
$Y_{2}$	.311	.310	.001	.002	1.0	.0	df	24							
$Y_{3}$	.747	.645	.102	.287	1.0	.0	RMSEA	.269							
$Y_{4}$	.833	.686	.146	.467	1.0	.0	CFI	.000							
$Y_{5}$	.543	.455	.088	.162	1.0	.0	AIC	20,381.394							
$Y_{6}$	.743	.693	.050	.162	1.0	0.0	BIC	20,505.587							

ML‐MSIT	Rel	Con	OSpe	$Rel^{w}$	IUSpe	ISpe	Model fit		$Cor(\theta_{i},\theta_{j})$						
**Scale**	**.949**	**.811**	**.138**	**.730**	**1.0**	**.0**	$\chi^{2}$	34.285		$\theta_{1}$	$\theta_{2}$	$\theta_{3}$	$\theta_{4}$	$\theta_{5}$	$\theta_{6}$
$Y_{1}$	.734	.633	.101	.276	1.0	.0	p	<.001	$\theta_{1}$						
$Y_{2}$	.712	.552	.038	.116	1.0	.0	df	9	$\theta_{2}$	.552					
$Y_{3}$	.819	.716	.103	.364	1.0	.0	RMSEA	.046	$\theta_{3}$	.833	.529				
$Y_{4}$	.860	.730	.130	.481	1.0	.0	CFI	.986	$\theta_{4}$	.882	.577	.924			
$Y_{5}$	.802	.685	.117	.371	1.0	.0	AIC	18,152.137	$\theta_{5}$	.746	.490	.745	.770		
$Y_{6}$	.817	.741	.075	.291	1.0	.0	BIC	18,353.951	$\theta_{6}$	.834	.629	.903	.897	.757	

ML‐MSST‐M‐1	Rel	Con	OSpe	$Rel^{w}$	IUSpe	ISpe	Model fit								
**Scale**	**.949**	**.811**	**.138**	**.730**	**.844**	**.156**	$\chi^{2}$	34.285							
$Y_{1}$	.734	.633	.101	.276	1.0	.0	p	<.001							
$Y_{2}$	.712	.675	.038	.116	.304	.696	df	9							
$Y_{3}$	.819	.716	.103	.364	.693	.307	RMSEA	.046							
$Y_{4}$	.860	.730	.130	.481	.779	.221	CFI	.986							
$Y_{5}$	.802	.685	.117	.371	.557	.443	AIC	18,152.137							
$Y_{6}$	.817	.714	.075	.291	.696	.304	BIC	18,353.951							

*Note*: ML‐MSST = Multilevel Multistate‐Singletrait Model, ML‐MSIT = Multilevel Multistate Indicator‐specific trait model, ML‐MSST‐M‐1 = Multistate‐Singletrait model with M‐1 method factors Rel = Reliability, Con = Consistency, Spe = Specificity, $Rel^{w}$ = Within‐subject reliability, IUSpe = Indicator‐unspecificity, ISpe = Indicator‐specificity, $Cov(\theta_{i},\theta_{j})$ = correlations between indicator‐specific trait variables. As the ML‐MSST‐M‐1 model is a reformulation of the ML‐MSIT model, both models result in identical model fit values when applied to the same data set. This is also true regarding the respective point estimates for the LST‐R and level‐specific coefficients, except for IUSpe and ISpe, which are always 1 and 0, respectively, in the ML‐MSIT model. Means, variances, and standard deviations of the sum scores of both scales are printed in bold.

## 4 GENERAL DISCUSSION

In the present study, we proposed and discussed the use of level‐specific coefficients (i.e. within‐ and between‐subject coefficients) besides the traditional (ML‐)‐LST coefficients reliability, consistency and occasion specificity, when conducting ML‐LST analyses. Furthermore, we showed that both the LST‐R and level‐specific coefficients can be estimated item‐ and scale‐wise (in contrast to previous work that only considered item‐wise reliability, consistency and occasion‐specificity (Castro‐Alvarez et al., 2022; Geiser et al., 2013; Steyer et al., 2015) and scale‐based within‐ and between‐level coefficients (Geldhof et al., 2014; Lai, 2021). We then compared estimates of these coefficients (both at the item and the scale level) obtained by the already established ML‐MSST and ML‐MSIT models with the ML‐MSST‐M‐1 LST model, a multilevel version of the LST model with M‐1 correlated method factors (Eid, 2000; Eid & Diener, 1999) that has, to our knowledge, not been presented in previous LST papers. We showed that, in all three models, estimates for within‐subject reliability coefficient substantially differed from those obtained for the occasion‐specificity coefficient and clarified their meaning in contrast to the occasion‐specificity coefficient. Additionally, we demonstrated that between‐subject “reliability” coefficients correspond to the indicator unspecificity (see also Koch and Semmler‐Busch, 2018; Eid et al., 2025) of the non‐reference items or unidimensionality of the entire scale.

When applying the ML‐MSST‐M‐1 model, the unidimensionality estimate for the entire ADM scale was rather high at .844 and comparable with ML‐CFA based values obtained in other studies using methodologically homogeneous scales (e.g. Frumkin et al., 2023; Heyde et al., 2023; Hachenberger et al., 2025). However, Item 2 showed a rather high indicator specificity, as implied by the low amount of variance that it shares with the reference item 1 (especially when compared to the indicator‐unspecificity of the other non‐reference items, whose shared variance with the reference item was notably larger). This is likely due to the wording of Item 2 (“Envied”), which possibly refers more to the rivalry facet of narcissism when compared to the other ADM items that are essentially synonyms for feelings of grandiosity (“Glorious”, “Prestigious”, “Brilliant”, “Powerful”, “Superior”; see Heyde et al., 2023), resulting in more homogeneous responses to the Items 1, 3, 4, 5 and 6. A further analysis of Rivalry scale, which we decided not to consider in our analyses due to their poor psychometric properties (see Appendix B), could be nonetheless interesting when item 2 from the ADM scale is included. This item does indeed sound more similar to the items of the rivalry scale (“Contemptuous”, “Irritated”, “Gleeful”, “Envious”, “Resentful”, “Angry”), at least at first glance, which could result in lower indicator specificity for this item when added to the rivalry scale.

Although occasion‐specificity estimates were substantially larger than 0 in all three models, the consistency of each questionnaire was notably higher than the occasion‐specificity (in each of the three models for each item, as well as the entire scale). Furthermore, occasion‐specificity estimates of this magnitude are typically observed in studies using trait measures as well (e.g. Deinzer et al., 1995), calling the proposed state‐level nature of the ADM construct into question (Heyde et al., 2023). Nevertheless, the within‐subject reliability of the ADM scale was notably large in the ML‐MSIT and the ML‐MSST‐M‐1 models with $Rel^{w}{\left(Y_{it}\right)}^{(ML-)MSIT}$ = $Rel^{w}{\left(Y_{it}\right)}^{(ML-)MSST-M-1}$ = .730, meaning that, despite a rather low occasion‐specificity of $OSpe{\left(Y_{it}\right)}^{(ML-)MSIT}$ = $OSpe{\left(Y_{it}\right)}^{(ML-)MSST-M-1}$ = .138, within‐person fluctuations as accounted for by the $Y_{it}^{w}$ variable can still be reliably captured by the latent state residual variable $\zeta_{t}$.

## 5 CONCLUSION

In the present study, we introduced the multilevel multistate‐singletrait model with M‐1 correlated method factors and demonstrated its overall good performance when applied on an ESM data set (Heyde et al., 2023). As an addition to the already existing ML‐MSIT model, this model is also capable of providing estimates of item‐ and scale‐wise between‐subject coefficients that can be meaningfully interpreted as the indicator‐(un)specificity of an item or the unidimensionality of a scale. Furthermore, we highlighted that the within‐level reliability coefficient can be of further use in ML‐LST analyses as it can be interpreted as a measure of the reliability of within‐person fluctations and, therefore, can be delineated from the occasion‐specificity coefficient (as a measure of the ratio of the latent state residual variance to the total variance of an item or a scale).

## AUTHOR CONTRIBUTIONS

**Lennart Nacke:** formal analysis; writing – review and editing; writing – original draft; visualization; software; validation; investigation; methodology. **Axel Mayer:** conceptualization; methodology; software; investigation; validation; formal analysis; writing – review and editing; writing – original draft; supervision.

## FUNDING INFORMATION

The authors received no specific funding for this work.

## CONFLICT OF INTEREST STATEMENT

We declare no conflicts of interest.

## DISCLOSURE OF ARTIFICIAL INTELLIGENCE‐GENERATED CONTENT (AIGC) TOOLS

The LaTeX code for the Equations ((14), (15), (16), (17)), as well as for Table 2, was generated with the help of GPT‐5.1.

## Figures and Tables

**FIGURE A1:**
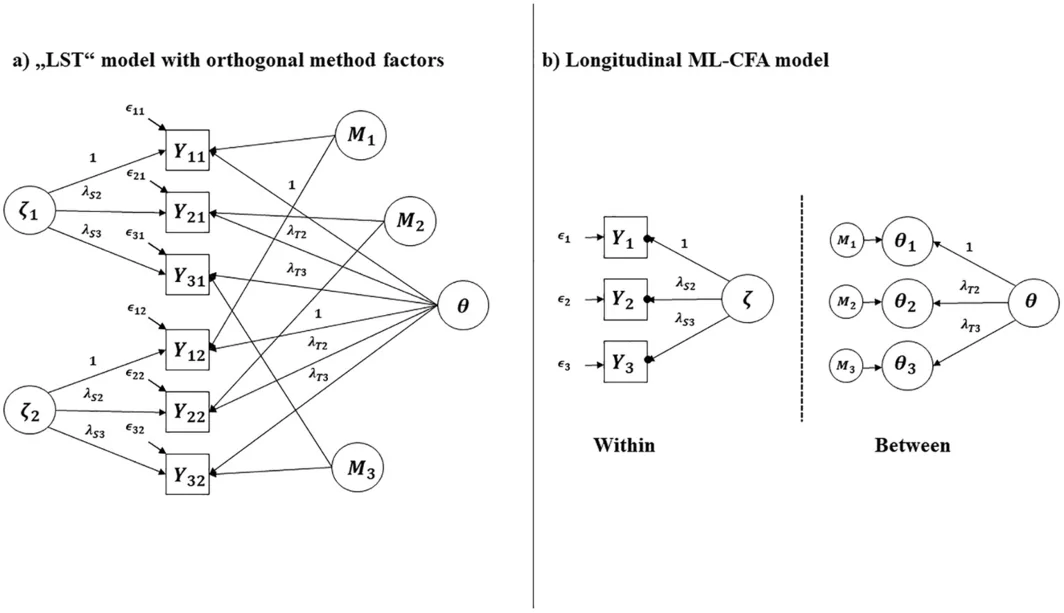
$Y_{it}$ = observed variable, θ = latent trait variable, $\zeta_{t}$ = latent state residual variable, $M_{i}$ = method factor, $\varepsilon_{it}$ = measurement error variable, $\lambda_{Ti}$ = trait loading coefficient, $\lambda_{Si}$ = state residual loading coefficient. Panel a) shows an “LST” model with orthogonal method factors (as it has been proposed by Steyer et al., 1992), whereas panel (b) depicts an longitudinal ML‐CFA model. The ML‐CFA model that has been proposed by Geldhof et al. (2014) as a method for obtaining level‐specific reliability coefficients is the multilevel specification of the LST model with orthogonal method factors when applied in a longitudinal research setting. However, since the orthogonal method factors or, in the ML‐CFA model, the between‐subject “error” variables, cannot be defined as conditional expectations of observed variables (Eid, 1996; Geiser & Lockhart, 2012), both models are not in line with LST‐R theory (Steyer et al., 2015). The loading coefficients of the method factors and residual variables are constrained to 1 which is not depicted to maintain readability. The variances of all latent variables and measurement error variables are held invariant accross time. The same is true for the loading coefficients $\lambda_{Ti}$ and $\lambda_{S1}$. Intercepts are not depicted due to readability and are also held invariant across time.

## Data Availability

The data for the empirical example are based on a study conducted on the situational fluctuations of narcissistic admiration and rivalry by Heyde et al. (2023) and are openly available at https://osf.io/srwbu/overview. The R code of all analyses is provided in an Open Science Framework repository (OSF; https://osf.io/47hkd/).

## Footnotes

^1^
In the ML‐CFA terminology used by Geldhof et al. (2014), the residuals at the between‐level are considered error variables. In turn, the coefficient quantifying the ratio of the variance that is shared by all indicators at the between‐level to the overall between‐level variance is called between‐subject “reliability”. However, from the perpective of LST‐R theory Steyer et al. (2015) measurement error variables should be modelled at level 1 as they are indicator‐ and timepoint‐specific. In turn, error variables at level 2 have not been defined in terms of this theory.

^2^
Similar multilevel versions of LST and related models that allow for the estimation of interpretable indicator‐specificity estimates have been proposed in the context of (cross‐sectional) Multitrait‐Multimethod (MTMM) and bifactor models (e.g. Eid et al., 2025; Koch & Semmler‐Busch, 2018).

3 In the remaining model equations pertaining to the ML‐MSIT and ML‐MSST‐M1 models, we also assume time‐invariance of the intercepts and factor loadings as this is a necessary constraint in ML‐LST analyses. However, note that all three LST models that are presented in this article can also be specified with non‐invariant factor loadings across time for the latent trait, state residual, and method‐specific variables (and time‐non‐invariant intercepts) when applied to wide‐formatted data.

^4^
The particulary low CFI value of .000 is due to the positive difference of the User Model $\chi^{2}$ value of 2326.310 and the Baseline Model $\chi^{2}$ that is 1900.550. This difference should be impossible or, at least, highly improbable, as it implies that a model assuming no relationships between the observed and latent components of the ML‐CFA model provides a better fit to the data as the ML‐MSST model. We suspect that this is the case since the ML‐MSST model assumes that the indicator‐specific trait variables $\theta_{i}$ are perfectly correlated with each other. In our example, this means that the model‐implied covariance matrix implies that 15 correlations at the between‐level are fixed to 1, which actually may be a more implausible assumption than assuming no correlations between these variables at all.

^5^
The loading coefficients of the method‐specific residual variables $M_{i}$ are fixed to 1 due to the assumption of measurement invariance across measurement occasions. However, note that these loadings may also bee freely estimated when the MSST‐M‐1 model is applied to a wide‐formatted data set (Geiser & Lockhart, 2012).

## APPENDIX B PRELIMINARY ML‐CFA Analyses OF THE ADM AND RIV SCALES

**TABLE B1:** ML‐CFA obtained fit estimates for the Admiration and the Rivalry scale.

Model fit: ADM		Model fit: RIV	
$\chi^{2}$	49.550	$\chi^{2}$	401.825
p	<.001	p	<.001
df	18	df	18
RMSEA	.037	RMSEA	.096
CFI	.983	CFI	.867
AIC	18,149.401	AIC	16,040.305
BIC	18,304.643	BIC	16,195.547

*Note*: ADM = narcisstic admiration, RIV = narcisstic rivalry. The model fit of each scale was evaluated using an ML‐CFA model (see Geldhof et al., 2014 and Figure A1). Due to its poor psychometric properties, the RIV scale was excluded from the ML‐LST analyses.
